# Screening the CALIBR ReFRAME Library in Search for Inhibitors of *Candida auris* Biofilm Formation

**DOI:** 10.3389/fcimb.2020.597931

**Published:** 2020-11-25

**Authors:** Gina Wall, Emily Chen, Mitchell V. Hull, Jose L. Lopez-Ribot

**Affiliations:** ^1^ Department of Biology and The South Texas Center for Emerging Infectious Diseases, The University of Texas at San Antonio, San Antonio, TX, United States; ^2^ Calibr, a division of The Scripps Research Institute, La Jolla, CA, United States

**Keywords:** *Candida auris*, antifungals, repurposing, drug screening, Calibr ReFRAME library

## Abstract

*Candida auris* is an emerging yeast which, since its first isolation about a decade ago, has spread rapidly and triggered major infectious outbreaks in health care facilities around the world. *C. auris* strains often display resistance to clinically-used antifungal agents, contributing to high mortality rates. Thus, there is an urgent need for new antifungals to contain the spread of this emerging multi-drug resistant pathogen and to improve patient outcomes. However, the timeline for the development of a new antifungal agent typically exceeds 10‑15 years. Thus, repurposing of current drugs could significantly accelerate the development and eventual deployment of novel therapies for the treatment of *C. auris* infections. Toward this end, in this study we have profiled a library of known drugs encompassing approximately 12,000 clinical-stage or FDA-approved small molecules in search for known molecules with antifungal activity against *C. auris*; more specifically, those capable of inhibiting *C. auris* biofilm formation. From this library, 100 compounds displaying antifungal activity were identified in the initial screen, including 26 compounds for which a dose-response relationship with biofilm-inhibitory activity against *C. auris* could be confirmed. Of these, five were identified as the most interesting potential repositionable candidates. Due to their known pharmacological and human safety profiles, identification of such compounds should allow for their accelerated preclinical and clinical development for the treatment of *C. auris* infections.

## Introduction

The opportunistic pathogenic yeast *C. auris* has recently expanded around the globe as a major cause of nosocomial outbreaks with high mortality rates (Chowdhary et al., 2016; Chowdhary et al., 2017; Lockhart et al., 2017; Kean et al., 2020). For example, in the United States, the first cases were reported just 4 years ago in 2016, with a total of 1,238 cases counted since then and an additional 2,397 patients colonized (CDC, 2019b). Clinical presentations and risk factors associated with *C. auris* infections are generally similar to those normally associated with candidiasis caused by other species. In general, three major features contribute to high mortality rates associated with these infections: *i)* *C. auris* appears to possess an unprecedented ability among known pathogenic fungi to easily spread between patients in healthcare facilities. This is likely related to its ability to colonize human skin where it can persist for long periods, to survive on environmental surfaces for several weeks, as well as to tolerate some of the most commonly used healthcare disinfectants (Cadnum et al., 2017; Piedrahita et al., 2017; Welsh et al., 2017; Kean et al., 2018; Ku et al., 2018). *ii)* The difficulty in correctly diagnosing *C. auris*, which in the past had often been misidentified as other close species (*i.e. C. haemulonii*) by clinical laboratories that use commercial systems such as VITEK 2 and API 20C AUX (Dewaele et al., 2019; Jackson et al., 2019), possibly causing incorrect treatment regimens to be instituted thereby allowing the infection to persist. *iii)* Particularly worrisome is the fact that strains of *C. auris* are often resistant to clinically-used antifungal drugs (Chowdhary et al., 2018). According to data from the Centers for Disease Control and Prevention (CDC), 90% of *C. auris* strains in the US have been resistant to fluconazole and 30% have been resistant to amphotericin B. Echinocandins generally retain potent *in vitro* activity against *C. auris* and are recommended as the first-line treatment by the CDC (CDC, 2018; Chowdhary et al., 2018). However, approximately 5% of *C. auris* clinical isolates in the US have been resistant to echinocandins. Indeed, a recent report from New York in 2019 described how *C. auris* strains from three different patients had developed pan-resistance during treatment with an echinocandin drug, and all three patients eventually succumbed to this infection (Ostrowsky et al., 2020).

Another factor that can contribute to pathogenesis is biofilm formation. *C. auris* isolates have been recovered from clinical sites including central venous catheters, stents, and wounds, and biofilm formation is known to confer increased resistance to antifungals (Larkin et al., 2017; Sherry et al., 2017; Rossato and Colombo, 2018; Romera et al., 2019). Besides its role in human infections, biofilm formation may also contribute to the ability of *C. auris* to survive on surfaces in the environment for long periods of time, which does facilitate its survival and persistence in healthcare facilities (Welsh et al., 2017; Ku et al., 2018).

Altogether, these unique characteristics have led to the emergence of *C. auris* as a major causative agent of serious outbreaks in healthcare settings, and as a result of these major concerns, in its recently released *Antibiotic Resistance Threats in the United States*, the CDC has designated *C. auris* as one of only 5 “Urgent Threats” demanding swift and aggressive action (CDC, 2019a).

Given the problems that could occur in the foreseeable future because of the low number of antifungals available, there is a clear need to investigate and identify new drugs to combat infections caused by *C. auris*. Drug repurposing or repositioning, the process of finding new therapeutic indications for existing drugs (Ashburn and Thor, 2004), represents a highly attractive approach which may potentially lead to the rapid development of drugs with novel antifungal activity against this new pathogenic fungus. For example, the FDA-approved anthelmintic niclosamide, after being first identified in a screen for inhibitors of *C. albicans* filamentation, was reported to inhibit *C. auris* biofilm formation (Garcia et al., 2018). Our group has previously screened both the Prestwick library and the Pathogen Box for inhibitors of this emerging yeast, whereas the Zaragoza group also screened the Prestwick library in search of off-patent drugs with novel antifungal activity against three different *C. auris* strains (Wall et al., 2018; de Oliveira et al., 2019; Wall et al., 2019). In this present study we have screened the ReFRAME (Repurposing, Focused Rescue, and Accelerated Medchem) library from Calibr at Scripps Research, in order to identify existing compounds with novel antifungal activity and efficacy to prevent biofilm formation by *C. auris*. This library contains approximately 12,000 compounds that consist of FDA-approved drugs, drugs in different stages of clinical development, and preclinical compounds with known pharmacokinetics, pharmacodynamics, and safety data in humans. This approach should allow for the accelerated development of potential therapies against infections caused by this emerging pathogenic yeast.

## Materials and Methods

### Strains and Culture Conditions

The *C. auris* isolate 0390 (South Asia, clade I) was used for all experiments performed. It was obtained from the U.S. Centers for Disease Control and Prevention (CDC) (CDC, 2019a). This isolate is resistant to azoles and amphotericin B, and shows reduced susceptibility to echinocandins according to the CDC.

Overnight cultures of *C. auris* 0390 were grown by inoculating 20 ml of yeast extract-peptone-dextrose (YPD) (1% [wt/vol] yeast extract, 2% [wt/vol] peptone, 2% [wt/vol] dextrose) liquid medium in 125-ml flasks and incubating in an orbital shaker (150 to 180 rpm) at 30°C. After 18 h, the cells were washed with phosphate-buffered saline (PBS) and counted with a hemocytometer. The cells were then adjusted to the desired final density (typically 2 x 10^6^ cells/ml for biofilm testing) in RPMI medium supplemented with _L_-glutamine (Cellgro, Manassas, VA) and buffered with 165 mM morpholinepropanesulfonic acid ([MOPS] Thermo-Fisher Scientific, Waltham, MA) at pH 6.9.

### Compound Library

The ReFRAME library (Janes et al., 2018) contains approximately 12,000 high-value molecules assembled by combining three databases (Clarivate Integrity, GVK Excelra GoStar and Citeline Pharmaprojects) for fast-track drug discovery. This library contains U.S. Food and Drug Administration (FDA)-approved/registered drugs (~35%), investigational new drugs at several stages of clinical development (~58%), and approximately 3% of preclinical compounds with available safety data (repeat dose efficacy or toxicity studies). Thus, a majority of the collection is comprised of non-approved therapeutics which offer excellent opportunities for repurposing. The compound solutions in DMSO were provided by Calibr to us in individual wells of 96-well flat-bottom microtiter plates (Corning Incorporated, Corning, NY), each in nanoliter volumes so that addition of the appropriate volume of cell suspension for the screening (see below) would result in a final concentration of each compound of 5 µM. The provided “assay-ready”, bar-coded plates were stored at -20°C until the screening was conducted.

### Primary Screen for Inhibitors of *C. auris* Biofilm Formation

The screen followed a similar method to what has been described by our group previously (Pierce et al., 2008), with minor modifications. The initial screen was performed against *C. auris* 0390 to identify inhibitors of biofilm formation. A total of 60 µl of the *C. auris* 0390 cell suspension at a concentration of 2 x 10^6^ cells/ml in RPMI medium were added to each well of the 96-well microtiter plates containing pre-spotted individual compounds of the ReFRAME library provided by Calibr. Wells in columns 1-10 contained individual compounds, which upon addition of the cell suspension, resulted in a final screening concentration of 5 µM; while wells in column 11 contained Amphotericin B (Gibco Life Technologies, Grand Island, NY) at 4 µg/ml as the positive control, and those in column 12 contained an equivalent amount of DMSO to serve as the growth control since all drug stocks were originally made in this solvent. After the addition of the cell suspension, the plates were incubated for 24 h at 37°C to allow for biofilm formation, and then the plates were washed with 150 µl of PBS to remove non-adherent cells. Finally, 100 µl of an XTT/menadione (Sigma, St. Louis, MO) solution were added to each well, incubated for 1h in the dark, and the biofilm inhibition was estimated by measuring the reduction of XTT by metabolically active cells in a plate reader as previously described by us (Pierce et al., 2008). Compounds found to inhibit greater than 40% of biofilm formation (based on XTT colorimetric readings) were selected as initial “hits.”

### Dose-Response Assays for Confirmation of Hits and Establishing Potency

**Figure 1:**
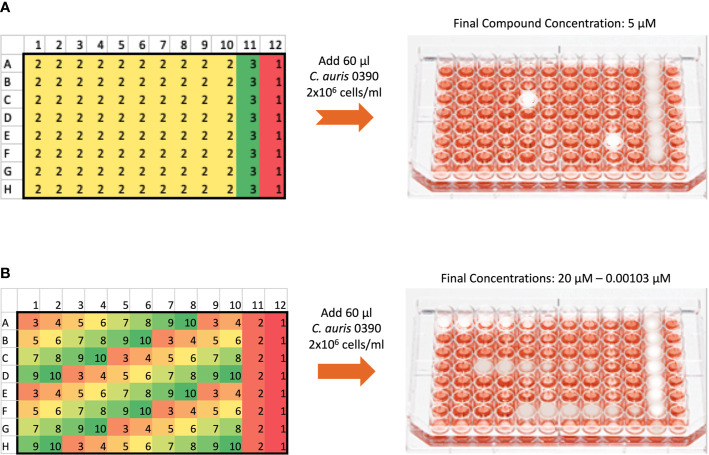
General methodology for the initial screen of the ReFRAME library and Dose-response assays using the 96-well microtiter plate model of *Candida* biofilm formation. **(A)** The initial screen was performed at a final concentration of 5 µM of each compound in the library. The plate map of the 96 well plates is as follows: 1 denotes wells with no compound to serve as growth controls for each plate, 2 denotes wells with test compounds, and 3 denotes wells with Amphotericin B at 4 µg/ml to serve as the positive control for inhibition. **(B)** The dose-response plates were used to confirm hits from the initial screen and establish their potency. Hits from the initial screen were tested in a series of concentrations ranging from 20 to 0.00103 µM. For each hit compound, serial dilutions are represented by the changing colors numbered 3 to 10, as seen for example for one hit compound from well A1 to A8. Wells labeled 2 in column 11 contained Amphotericin B at a final concentration of 4 µg/ml, and wells labeled 1 in column 12 contained no compound to serve as growth controls.

The dose-response assays were carried out using the same 96-well microtiter plate model for inhibition of *C. auris* biofilm formation using an eight-point dose-response experiment of each initial hit compound, with final concentrations ranging from 20 to 0.00103 µM (**Figure 1**). The plates were processed as described above, and the percent inhibition was calculated using the XTT-reduction colorimetric assay. From these results, the inhibitory concentration required to inhibit 50% of growth (IC_50_) was determined by fitting normalized results (positive (untreated) and negative (uninoculated) controls arbitrarily set as 100% and 0% growth) to the variable slope Hill equation (an equation that determines the nonlinear drug dose-response relationship) using Prism (GraphPad Software Inc., San Diego, CA). Compounds found to inhibit greater than 50% of biofilm growth at 20 µM or lower concentrations were considered to be confirmed “hits.”

### Data Availability

Additional information for assay description, methodology, experimental data and hit compounds are available in the ReFrame database, an open and extendable drug repurposing data, accessible through https://reframedb.org/assays/A00274.

## Results

### Screening the ReFRAME Library for Inhibitors of *C. auris* Biofilm Formation

**Figure 2:**
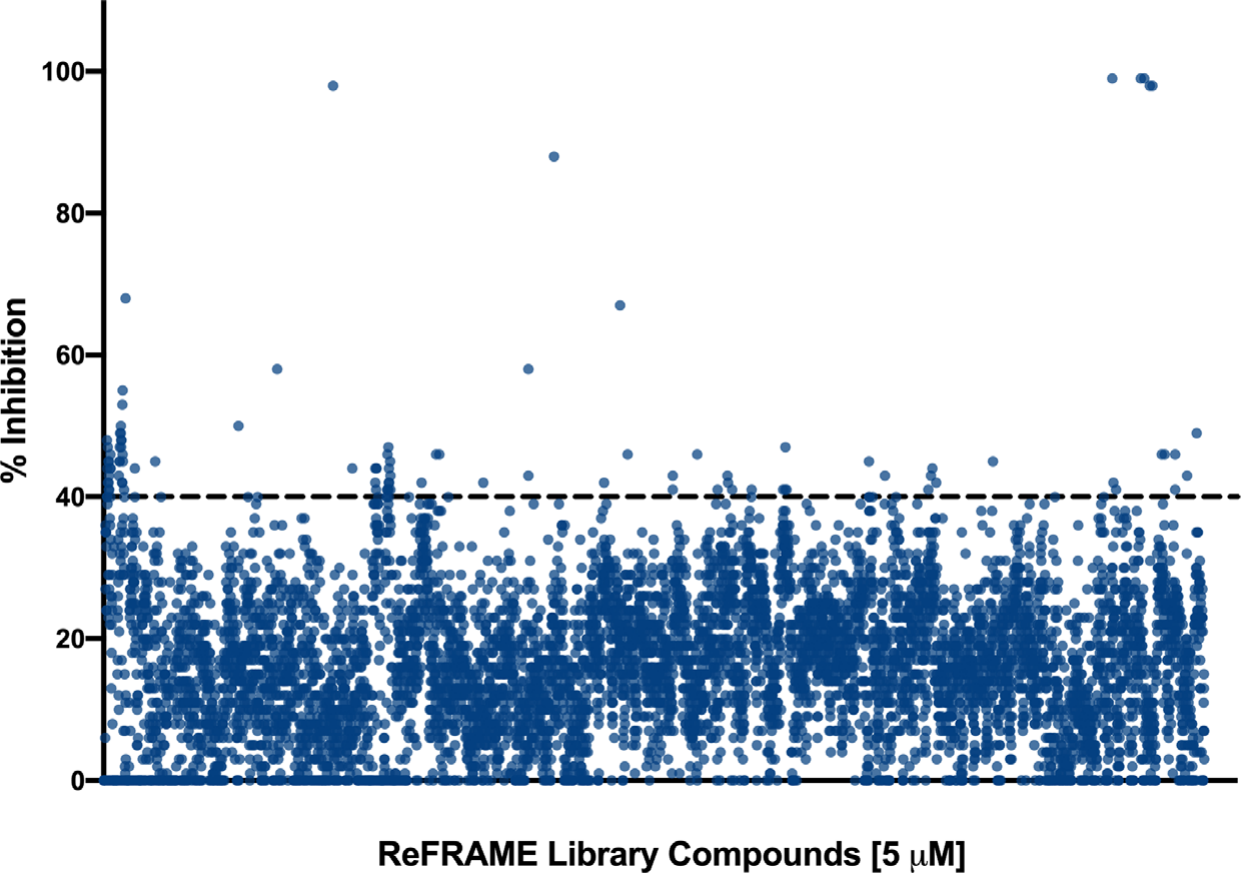
Graphical representation of the initial screen of the Calibr ReFRAME library in search for compounds with inhibitory activity against *C. auris* strain 0390. The threshold was arbitrarily set at 40%.

We performed a primary screen of the ReFRAME library at 5 µM to identify inhibitors of *C. auris* biofilm formation. The screen was performed using the 96-well plate biofilm formation method described previously by our lab (Pierce et al., 2008), with some minor modifications. The drugs were provided to us by Calibr pre-spotted in wells of the microtiter plates, and our normal procedure was slightly altered to use 60 µl of cells rather than 100 µl in order to allow for less compound to be used in the screen, also as described in materials and methods. The screen was performed using *C. auris* 0390 because among strains in the CDC panel, it is the least susceptible to clinically-used antifungals (CDC, 2019a). We chose to screen this library for inhibitors of *C. auris* biofilm formation because there is evidence that *C. auris* has the ability to form biofilms, and it is known that biofilm formation contributes to the mortality seen in these infections (Sherry et al., 2017). The plates were read after 24 h incubation (to allow for biofilm formation). After normalization of the data, given the low concentration (5 µM) at which the screening was performed, the initial cutoff for “hits” was arbitrarily set relatively low at 40%. Therefore, a “hit” was defined as a compound that inhibited at least 40% or more of the biofilm formation of *C. auris* under the conditions of our assay. Using this criterion, a total of 100 compounds were initially classified as “hits” (**Figure 2**), resulting in an initial hit rate of 0.83%.

### Dose-Response Assays to Confirm Activity and Determine the Potency of Initial Hits

Since the initial screen was performed in a blinded fashion (the identity of the compounds was not provided to us at the time of the screen), the ID numbers for wells where >40% inhibition was detected in each bar-coded plate were provided to Calibr so that the “hit” compounds could be identified by the organization in the ReFRAME database. Then, dose-response plates containing 8 different dilutions for each initial hit compound were prepared and provided to us by Calibr, as described in Materials and Methods, and dose-response assays were performed for confirmatory purposes, and at the same time to establish the potency of any confirmed hit compound. From results of these assays, we considered a hit to be confirmed if its inhibitory activity at the highest concentration of 20 µM or lower was at least 50%. Using this criterion, 26 out of the original 100 hit compounds were confirmed. These results were sent to Calibr, which then provided the identity of the confirmed hits. For each confirmed compound, **Table 1** shows the drug name, its efficacy (or highest level of inhibition achieved in the dose-response assays), as well as the calculated IC_50_ value. As expected, a majority of them were known antifungals or antiseptics, thereby providing reassurance to the validity of our assay. In addition, 5 compounds were classified as potential repurposing “hits” (**Table 1**). These were Tazomeline, Lonafarnib, AM-24 (2,4,6-triiodophenol), Miltefosine, and Provecta (rose bengal disodium).

**Table 1:** Identity, efficacy, and potency of the 26 confirmed hits from dose-response assays.

Drug Class (underlined) and Name	Maximum % Inhibition from Dose-Response Assays	IC_50_ (µM) from Dose Response Assays
Antifungals/Fungicides		
Posaconazole	65%	1.854
Anidulafungin	101%	0.108
Anidulafungin	101%	3.12
Anidulafungin	97%	0.2541
Isavuconazole	74%	6.409
DuP-860	72%	10.23
Ketoconazole	73%	5.392
Fludioxonil	63%	24.77
Micafungin Sodium	97%	0.2028
E-1210 (active moiety of prodrug APX001, Fosmanogepix)	100%	0.1277
Sirolimus	97%	1.637
Buclosamide	50%	26.97
VT-1161	55%	14.63
Ravuconazole	67%	4.203
WF11899A (FR901379, precursor of micafungin)	100%	0.03324
Antiseptics/Disinfectants/Antibacterials		
Octenidine	101%	2.647
Chelerythrine	59%	10.23
Gentian violet	99%	2.98
Formaldehyde	100%	0.20970
Phenylmercuric borate	102%	1.305
Cycloheximide	85%	0.863
Repositionable candidates		
Tazomeline	80%	4.646
Lonafarnib	60%	13.35
AM-24	80%	4.772
Miltefosine	100%	8.006
Provecta	50%	17.61

## Discussion

Since its first identification as a causative agent of infection in humans in 2009, *Candida auris* has emerged as a formidable opportunistic pathogenic yeast and rapidly spread throughout the world (Chowdhary et al., 2016; Chowdhary et al., 2017; Lockhart et al., 2017; Kean et al., 2020). *C. auris* is able to live on inert surfaces in the environment as well as on human skin for an extended period of time, increasing its chance to spread from patient to patient (Ku et al., 2018; Kean et al., 2020). *C. auris* is also known to be intrinsically resistant to fluconazole, the most commonly used azole antifungal agent, and multidrug resistance is being increasingly detected in strains from different geographical locations, leading to high mortality rates (Chowdhary et al., 2018). Biofilm formation contributes to environmental persistence and resistance to treatment (Larkin et al., 2017; Sherry et al., 2017). Clearly, new management strategies are needed to contain the spread of this emerging pathogen and to improve patient outcomes.

Drug repurposing (also referred to as drug repositioning) consists of the re‐application of known drugs to target new therapeutic indication(s); whereas drug rescuing refers to situations where a drug candidate initially failed for its purported indication, but can be conceivably “rescued” for a completely different therapeutic indication. Initiating drug discovery campaigns from known drugs, or from advanced compounds with optimized pharmacokinetics and safety (collectively, ’high value-added compounds’), significantly reduces the burden associated with finding new therapies (Nosengo, 2016). These efforts can be facilitated by screening repurposing libraries with high representation of FDA-approved compounds. Indeed, in the last few years there has been an increasing interest by different academic groups in the screening of such libraries in search for drugs with novel antifungal activity (recently reviewed in (Wall and Lopez-Ribot, 2020b). In the case of *C. auris*, there is only a limited number of reports regarding the implementation of this approach (Wall et al., 2018; de Oliveira et al., 2019; Wall et al., 2019). However, a comprehensive collection of such compounds has been missing from the chemical libraries of most commercial and nonprofit drug-discovery organizations; or if existed, has not been widely available to researchers in academia. In particular, drugs that have been tested in clinical trials but did not reach approval are not readily available, which complicates screening efforts. To address this critical gap and overcome these major hurdles, Calibr, a division of The Scripps Research Institute, which was established in 2012 with the goal of accelerating the translation of basic research to new medicines that address unmet medical needs, recently assembled the ReFRAME library (Janes et al., 2018). This library consists of approximately 12,000 high-value compounds. Compounds are eligible for inclusion in this library based on a number of criteria: i) chemical structure and key data are in the public domain, ii) reasonable annotation of dosing and safety in humans or repeat dosing in animals, and iii) privileged pharmacology (i.e., compounds are generally cell-permeable, non-cytotoxic, and metabolically stable). Overall, the ReFRAME library is truly unique among repurposing collections because of its scale and comprehensiveness. Importantly, there is a high degree of structural diversity within the library and the collection is sufficiently representative of the majority of chemotypes that have reached clinical development. It follows that hits from the ReFRAME library screening are either known drugs or molecules far along in the drug discovery process; thus, these molecules can form the basis for rapid generation of a clinical candidate.

Because our initial screen still used a 96-well microtiter plate model (Pierce et al., 2008), we were limited by the amount of compound which could be pre-spotted in each well, considering the larger volume per well as compared to true high throughput screening formats typically based on the use of 384-well (or even higher density) plates. In any case, we used a lower total volume as compared to our normal methodology (60 versus 100 microliters) in order to use less quantity of each compound. Because of these considerations, we screened the library at a relatively low concentration of 5 µM, as compared to our previous efforts that have typically used 10–20 µM concentrations (Wall et al., 2018; Wall et al., 2019). After performing the initial screen, given this low screening concentration, and also considering the fact that the screen was performed under biofilm-growing conditions under which *C. auris* is known to display intrinsic resistance to treatment (Sherry et al., 2017), we arbitrarily set the initial cutoff for determining hit compounds at a relatively low threshold of 40% inhibition (**Figure 1**). This resulted in a manageable number of 100 initial hits, although we fully realized that many of them may represent false positives that would not be confirmed in subsequent dose-response experiments. Indeed this was the case, and only 26 of the 100 initial hit compounds (or about one fourth) displayed dose-response inhibitory activity in follow-up confirmatory experiments (**Table 1**).

Once the identity of these compounds was revealed (the primary screen was performed in a blinded fashion), not surprisingly and providing validation to the robustness of our assay, a majority of confirmed hits were known antifungals and antiseptics (see **Table 1**). Although of limited value from a repurposing perspective, this information may be important for the clinical management of patients and prevention of outbreaks. For example, the identification of different antiseptics can be important for the disinfection of surfaces where formation of environmental *C. auris* biofilms can lead to its persistence and contribute to outbreaks (Ku et al., 2018). In respect to known antifungals, several azoles and echinocandins were identified and confirmed in our assays with the ability to inhibit biofilm formation by *C. auris*. Although preformed biofilms are intrinsically resistant to these two classes of antifungals, our results indicate that they could be used to prevent biofilm formation. Of note, WF11899A, which happens to be the precursor of micafungin, displays the most potent activity (about 30 nM) of all compounds tested to date in our laboratory; however during its development its direct use was prevented by its high levels of hemolytic activity (Hashimoto, 2009). Also of interest was the inhibitory activity detected in the cases of E-1210 and VT-1161, two novel antifungals currently undergoing clinical trials (Wall and Lopez-Ribot, 2020a).

Besides known antifungals and antiseptics, from a repurposing point of view our experiments confirmed the antifungal activity associated with 5 different compounds representing potential repositionable drugs (**Table 1**). These were Tazomeline, Lonafarnib, AM-24 (2,4,6-triiodophenol), Miltefosine, and Provecta. Each of the five repurposing compounds identified in this screen have different modes of action in their original clinical indications. Tazomeline is a muscarinic M1 agonist, and when M1, an acetylcholine receptor, is activated, it has been shown to enhance cognition in mice (Eglen et al., 1999). This drug has been through Phase II clinical trials for treatment of Alzheimer’s disease, however there has not been progression into Phase III. Lonafarnib inhibits farnesyl transferase, which is involved with lipid modification for translated proteins (Moorthy et al., 2013), and has undergone phase II clinical trials for the treatment of progeria and hepatitis delta virus (HDV) infections (Wong and Morse, 2012; Biopharmaceuticals, 2018). This compound has also been granted Orphan Drug Designation and Fast Track Designation by the FDA (Biopharmaceuticals, 2018). AM-24 or 2,4,6-triiodophenol, is known to be a lipoxygenase inhibitor which seems to inhibit synthesis of leukotriene B_4_ (Trocóniz et al., 2006). This drug has been through phase I clinical trials to assess its efficacy and safety for enzyme inhibition in humans; however no further clinical trials seem to have been performed at this time (Trocóniz et al., 2006). Miltefosine is an alkylphosphocholine drug thought to inhibit protein kinase involved in cell survival (Dorlo et al., 2012). It is currently used to treat visceral and cutaneous leishmaniasis, and this compound has been previously identified by our laboratory and others as having efficacy against *C. albicans* and *C. auris* biofilms (Dorlo et al., 2012; Vila et al., 2015; Wall et al., 2019). Provecta (rose bengal disodium) is currently used to prevent and treat flea infestations in animals, mainly cats and dogs (Liszka and Dhang, 2014), while a different formulation is being evaluated for the treatment for certain cancers, skin conditions and other infections (Vanerio et al., 2019).

Overall our screen of the ReFRAME library, to our knowledge the largest repurposing library currently assembled, resulted in the identification of different known antifungals with activity against *C. auris*, as well as several antiseptics that may play a role in controlling outbreaks by preventing or limiting its environmental persistence in health care settings. From a repurposing point of view, the 5 potentially repositionable candidates may offer much needed alternatives for the treatment of these infections. However, much further work is required to confirm their antifungal activity against *C. auris*, and potentially other pathogenic fungi. For example, key toxicological and pharmacokinetic/pharmacodynamic properties of the repurposed compounds for its new intended use as antifungals need to be taken in consideration, and their *in vivo* activity will need to be determined in clinically-relevant models of infections. In any case, this screen shows the effectiveness of a drug repurposing approach in identifying potential molecules with antifungal activity, which optimistically could be quickly deployed in the near future for the treatment of these devastating infections.

## Data Availability Statement

The datasets presented in this study can be found in online repositories. The names of the repository/repositories and accession number(s) can be found below: Data are available through https://reframedb.org (assay A00274).

## Author Contributions

Conceptualization: JL-R and GW. Methodology: JL-R, GW, EC, and MH. Experimentation: GW, EC, MH. Data Analysis: GW, EC, MH, and JL-R. Original Draft Preparation: GW. Writing—Review and Editing: GW, EC, MH, and JL-R. Funding Acquisition: JL-R. All authors contributed to the article and approved the submitted version.

## Funding

This work was supported by NIH grant R21AI140823 from the National Institute of Allergy and Infectious Diseases to JL-R. Additional support was provided by the Margaret Batts Tobin Foundation, San Antonio, TX. GW was the recipient of a Science, Mathematics, and Research for Transformation (SMART) fellowship provided by the Department of Defense. The funders had no role in study design, data collection and analysis, decision to publish, or preparation of the manuscript, and the content is solely the responsibility of the authors.

## Conflict of Interest

The authors declare that the research was conducted in the absence of any commercial or financial relationships that could be construed as a potential conflict of interest.

## Supplementary Material

Supplementary Figure 1 Chemical structures of the 5 potential repositionable compounds.
