# Supplementary figures and images for: Screening the CALIBR ReFRAME Library in Search for Inhibitors of Candida auris Biofilm Formation
Source: Front Cell Infect Microbiol. 2020 Nov 25;10:597931. doi: 10.3389/fcimb.2020.597931 (PMC7723901; doi:10.3389/fcimb.2020.597931)

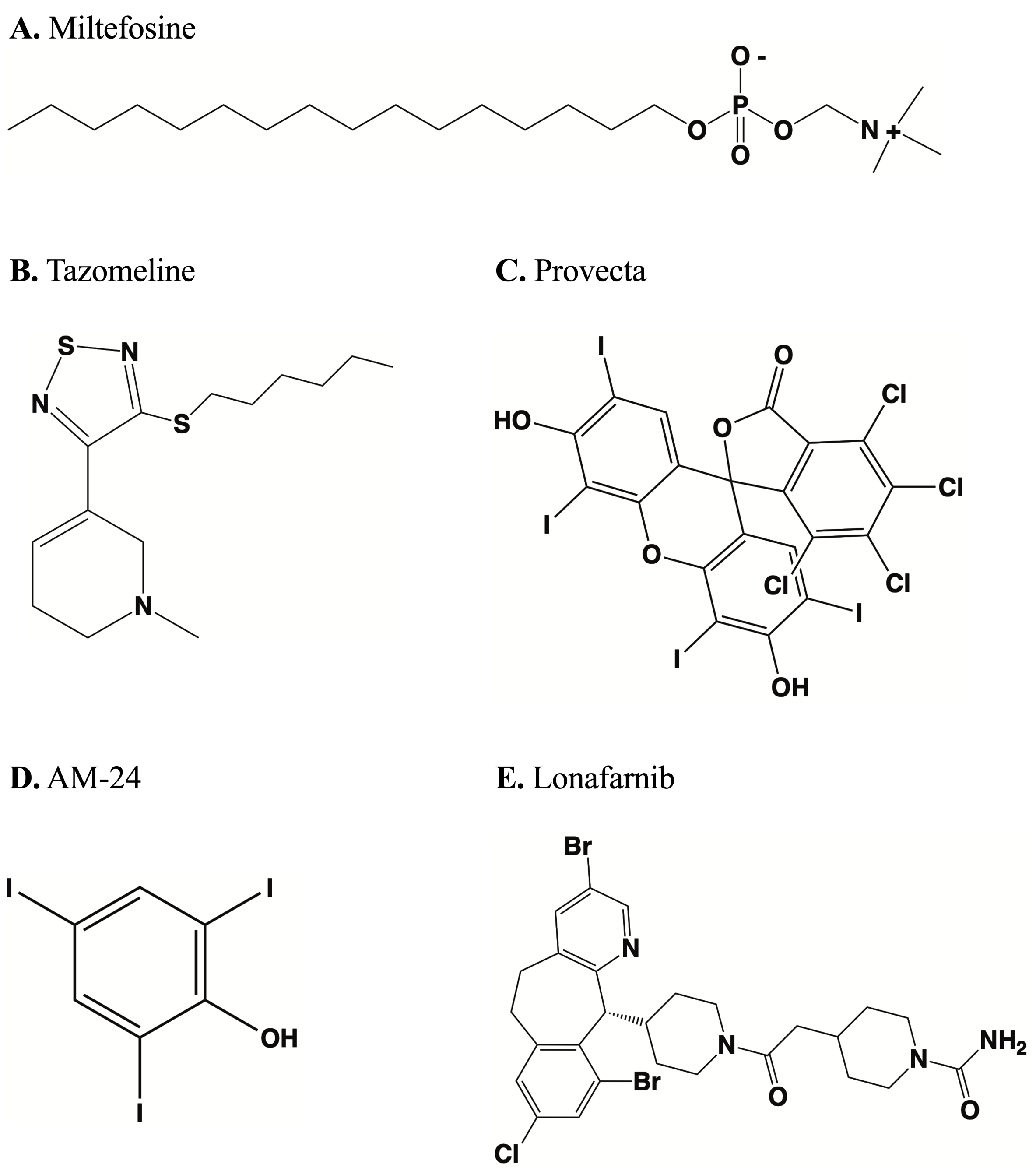

Supplement: Supplementary Figure 1 — Chemical structures of the 5 potential repositionable compounds. [file Image_1.tif]
